# Exposures Before Issuance of Stay-at-Home Orders Among Persons with Laboratory-Confirmed COVID-19 — Colorado, March 2020

**DOI:** 10.15585/mmwr.mm6926e4

**Published:** 2020-07-03

**Authors:** Kristen Marshall, Grace M. Vahey, Emily McDonald, Jacqueline E. Tate, Rachel Herlihy, Claire M. Midgley, Breanna Kawasaki, Marie E. Killerby, Nisha B. Alden, J. Erin Staples, Colorado Investigation Team, Colorado Investigation Team, Alison J. Basile, Alyssa R. Beck, Karen L. Boroughs, Paul L. Burns, Cathy L. Buschmeier, Nathaniel M. Byers, Amanda E. Calvert, Trudy V. Chambers, David T. Dennis, Mary Ellen Fernandez, Katherine T. Ficalora, Kelly A. Fitzpatrick, Shannon Fleck-Derderian, Erik S. Foster, Christin H. Goodman, Garrett Heck, Claire Y-H. Huang, Amy J. Lambert, Aine Lehane, Jennifer A. Lehman, Kristine Lindell, Nicole P. Lindsey, Sarah E. Maes, Courtney Nawrocki, Nancy H. Nay, Kathleen A. Orloski, Lynn Osikowicz, Christina Parise, Lara C. Perinet, Mark A. Pilgard, Jordan A. Powers, María F. Rizzo, Brandy J. Russell, Tracey M. Semcer, Benjamin Skinner, Melanie Spillane

**Affiliations:** ^1^CDC COVID-19 Emergency Response; ^2^Colorado Department of Public Health and Environment; ^3^Epidemic Intelligence Service, CDC.; CDC; CDC; CDC; CDC; CDC; CDC; CDC; CDC; CDC; CDC; CDC; CDC; CDC; CDC; CDC; CDC; CDC; CDC; CDC; CDC; CDC; CDC; CDC; CDC; CDC; CDC; CDC; CDC; CDC; CDC; CDC; CDC; CDC; CDC; CDC; CDC.

On March 26, 2020, Colorado instituted stay-at-home orders to reduce community transmission of SARS-CoV-2, the virus that causes coronavirus disease 2019 (COVID-19). To inform public health messaging and measures that could be used after reopening, persons with laboratory-confirmed COVID-19 during March 9–26 from nine Colorado counties comprising approximately 80% of the state's population^†^ (Adams, Arapahoe, Boulder, Denver, Douglas, El Paso, Jefferson, Larimer, and Weld) were asked about possible exposures to SARS-CoV-2 before implementation of stay-at-home orders. Among 1,738 persons meeting the inclusion criteria^§^ in the Colorado Electronic Disease Surveillance System, 600 were randomly selected and interviewed using a standardized questionnaire by telephone. Data collection during April 10–30 included information about demographic characteristics, occupations, and selected activities in the 2 weeks preceding symptom onset. During the period examined, SARS-CoV-2 molecular testing was widely available in Colorado; community transmission was documented before implementation of the stay-at-home order. At least three attempts were made to contact all selected patients or their proxy (for deceased patients, minors, and persons unable to be interviewed [e.g., those with dementia]) on at least 2 separate days, at different times of day. Data were entered into a Research Electronic Data Capture (version 9.5.13; Vanderbilt University) database, and descriptive analyses used R statistical software (version 3.6.3; The R Foundation).

Among the 600 randomly selected COVID-19 patients, 133 (22%) were unreachable, 57 (10%) declined to participate, and 46 (8%) were ineligible (e.g., the onset date was too early or the patient was asymptomatic), leaving 364 (61%) participants. The median age of participants was 50 years (interquartile range = 34–64 years), and 187 (51%) were male. Overall, 206 (57%) participants identified as non-Hispanic white and 75 (21%) as Hispanic. Among all participants, 345 (95%) reported having health insurance, 128 (35%) were hospitalized and 18 (5%) died. Occupations reported by the 264 (73%) working participants were most frequently categorized into the following workplace settings^¶^: health care (99; 38%), professional or office setting (46; 17%), public administration or armed forces (18; 7%), and manufacturing (including meat-packing) (15; 6%).

Among all participants, 99 (27%) reported known contact with at least one person with laboratory-confirmed COVID-19 (Figure); the most commonly reported relationships to potential source patients were a family member (27; 27%) and a coworker (25; 25%). Approximately three quarters of participants reported that their exposure to a known COVID-19 contact occurred in the workplace (47; 47%) or the household (24; 24%). Among the 47 participants who reported workplace exposure, most were health care personnel (28; 60%), followed by workers in public administration or the armed forces (six; 13%), and those working in a manufacturing setting (five; 11%).

**FIGURE:**
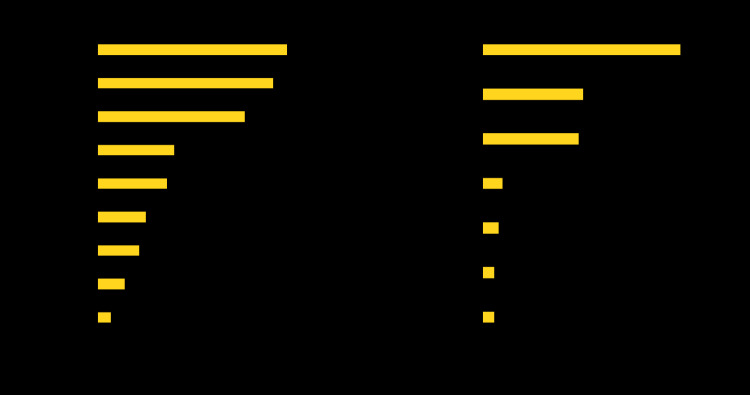
Reported relationships* and settings^†^ of exposure to persons with laboratory-confirmed COVID-19 among persons (N = 99) infected before institution of stay-at-home orders^§^ — Colorado, March 2020 **Abbreviation:** COVID-19 = coronavirus disease 2019. * Responses to exposure relationship and setting were not mutually exclusive. Family members include spouse (14%) and parent, child, or other family member (13%). ^†^ Health care personnel reporting exposure at work were classified as having workplace exposure. ^§^ March 26, 2020.

Among the 265 (73%) participants without known contact with a laboratory-confirmed COVID-19 patient, 30% (79 of 265) reported contact with a person they knew who had fever or respiratory symptoms. The most commonly reported activities in the 2 weeks before becoming ill included attending gatherings of >10 persons (116; 44%), traveling domestically (76; 29%), working in a health care setting (75; 28%), visiting a health care setting not as a health care worker (61; 23%), and using public transportation (57; 22%).

These findings highlight the need for anyone with COVID-19–compatible symptoms to avoid public settings and isolate from other persons, even within their own household, when possible (*1*,*2*). Because workplaces are common locations of potential exposure to persons with COVID-19, it is important that company officials and managers refer to CDC’s guidance for workplaces during the COVID-19 pandemic to minimize risk for exposure for their employees and customers (*3*). To protect their employees, patients, and other persons who enter their facilities, managers and staff members of health care facilities are encouraged to continue to follow CDC infection prevention and control practices (*4*). Because approximately one half of participants did not report contact with either a confirmed COVID-19 case or someone they knew with fever or respiratory symptoms, hand hygiene, social distancing, and wearing face coverings remain important strategies to practice while SARS-CoV-2 continues to circulate (*5*).

The findings in this report are subject to at least three limitations. First, this analysis did not include a comparison group of persons without COVID-19; thus, these findings are descriptive. Second, these findings are likely not generalizable to other populations because of potential response bias and differences in age distribution, disease severity, testing practices, or socioeconomic status between participants in this investigation and other populations. Finally, other community mitigation interventions, such as restrictions on gatherings of ≥50 persons, had been implemented before the stay-at-home orders were issued in Colorado, which likely also affected reported activities and potential exposure locations.

Depending on local guidance and circumstances, health departments should consider prioritizing case investigation and contact tracing to ensure prompt notification of exposed contacts. As jurisdictions continue reopening, mitigation strategies will need to be reviewed and potentially augmented to reduce the spread of SARS-CoV-2.

## Footnotes

* These authors contributed equally.

†
https://demography.dola.colorado.gov/population/population-totals-counties/.

§ Inclusion criteria consisted of laboratory-confirmed SARS-CoV-2 infection, presence of ≥1 symptom to establish illness onset date, and known hospitalization status.

¶ Occupations were coded using the Census Industry and Occupation Classification System (https://www.cdc.gov/niosh/topics/coding/code.html and https://wwwn.cdc.gov/nioccs3/Default.aspx).
